# Supplementary material for: Pleiotropic Functions of FoxN1: Regulating Different Target Genes during Embryogenesis and Nymph Molting in the Brown Planthopper
Source: Int J Mol Sci. 2020 Jun 13;21(12):4222. doi: 10.3390/ijms21124222 (PMC7353072; doi:10.3390/ijms21124222)
Supplement: Supplementary file 1 [file ijms-21-04222-s001.pdf]

International Journal of
Molecular Sciences

MDPI

Table S1. The primers and RNAi efficiency in this study. The primers used for qPCR, RNAi are highlighted in red and yellow, respectively. The primers used for RNAi targeting the second non-overlapping region are highlighted in green. The sequences of FHD were boxed.

>NlFoxN1

ATGGATATGTACTTAAGCCCTCCTGACAGTTTCAATCTGCAGGAGATGCTCGATTGTGATAT
CAAATGTGAGATGGCAGATGGGTTCGGCCTCGACTGCGACCTACCGCCCCTCGACCTAGAG
GATGAATCACACCACGCGTGGATCCATGGCAACTCGAACTCTAGTTTCGAGTTGGACTTTTT
TGGCACCGACTCGAGCGCCTCACTCATGGTCAATCCGCACGCCATCATGCCCCTACCCTCC
CTGCAGCACCTCAGCACAATTCGATCAGCTGATGTCGGCAGTCGATCAGCTGATCTCGGCA
GCTGTGACGTCATCAAGGTCGAACTCCCCTCCACGCCGCCATCACCCGATGATCGGTCTGA
CCATTTGATGGGAGACGACGAACCAGCAGAAGAGGAAGAAGAAGAAGACAACGAAGAG
GAAGAACCGGAAGACGATGATGATGATGAGGACTACCAGGAAGAGAGGAAGCCAAAGTC
GTTGAGAGTGGATCCCCTGAAAGTGTCACTAAGTGCCAGCTCAGTGGCAAGCAAGCAGCT
GGCCAGTATCACTGCCAACGCCAACGCCAACACACCCAACGGCTCAATCAAGAGTGAGAC
TGCTGCCAGTCCTTACACACCCCCCACCACTACCAAGAGTAAGAGGCCCATCGTGAGTTCG
AGAAAACAGCTGTGTCTAAATGATGCCAATCTCTATCCGAAGCCAGCCTACTCGTATTCGT
GTCTTATAGCCTTGGCTTTGAAAAATTCACCGTCTGGAAGTTTACCCGTTTCAGAAATCTAC
AATTTTATGTGCAAGCACTTTCCGTACTTCAAGTCGGCGCCGAACGGCTGGAAGAATTCGG
TGCGCCACAACCTGTCGCTCAACAAGTGCTTCGAGAAGATCGAGAAGCCGATGGCGGGCG
GTGAGGGGGCCGGAGGGGGGTCACAGCGAAAAGGCTGTCTGTGGGCGATGAACCCGCAC
CGGCTGGCCAAGATGGACGACGAAGTGGCCAAGTGGTCGCGCAAGGATCCGTTGGCGATA
CGCCGGTCTATGCTCAATCCAGATGACCTGGAGTCGCTGGAACGAGGAGATTTGCACGTGG
TCTGGATACCCGGTGACGGGTGTGGGGAGGGAGATGACTCGACAGGGGAGGACCAAGATG
GCGGCGCTCGTGACCCCCTAATGGCCGCCACTGCTCCCCCCGCCACCGCCCCCGCAGTGCA
GCCGCAGCCCAAAGTGGCGCCAATCAGGATTAGGACGGTAGCGGCCAAAGTTAGACCGCA
ACCTACCGCAACTAGCACAGTAACCGCCACAACCGCAGCTGAGGTTCTAAGACAAGAAGT
AGTTCACCAGCCATCACACGTAGTTAAACACGAAGTGGTGGAACACCACGATTACGAAGA
GGAAGAGGAGGAAGCGGAGGAGGAGGAGGATGAAGATGAGGATGAGGACGAAGAGGAG
GAGGAAGAGGAAGAAGAAGAAGAGGAGGAAGAGGAGGAGGAGGATGATGGGCCTCAGC
CGATTGGGAGTGATGAGAGTGAGGATGAGTTGAGCATGATGCCCCCCTCCCTGCAGCCTTT
CGAGCCCAGGTTGGCACTGGCCAAGAAGTTTACCGAAGCTCAGAAGGAGTTGAGGTTAGG
TAGCGGTGGGCAGCAGCATGATATCGAGTTGACAAACGACATCCTTGAATCACTGAACTGG
GTGGATCTTCCGCGCTACAAGTCGCCCGGCTCCAGGTCGCTGGACGTGACCGTGCCTGTCTC
CGACTACTCGGACGTGTACGCGGCCAACGTGCGAACGATCGCCACCGCGGCGAATGTGCG
AACGATCGCGAACGTGCGAACAATCGCCACCGCCCCCGGCGTGGTCGCCGCCTCCCGCAA
GCGTCCCGCCCCCACACGCATACAGGGCAACTACGTCTGTGTTAAGCGCAAACTGCCTCAG
GTTAACTGA

Primers for *NlFoxN1*.

Primer usage	Direction	Primer sequence (5′–3′)	RNAi efficiency (%)
qPCR	F	CAAGATGGCGGCGCTCGTGAC	
	R	AGCTGCGGTTGTGGCGGTTACTGT	
RNAi	F1	T7-AGTGGCAAGCAAGCAGC	92.05 ± 2.82
	R1	T7-GGTTCATCGCCCACAGA	
	F2	T7-AGGAGGATGAAGATGAGGAT	90.02 ± 4.40
	R2	T7-GCTTAACACAGACGTAGTTG	

>chr02.0345

ATGGAGAATCATGATACTTCTGCAAAAGCTGAAGAAAATATTGATTGTAATGGCAAGAAG
GAAGAAGAGGCGGAAGAAGAAGACGACGGTGATGATGATGATGAAGACTCACTGTCGGA
GGAAGTGGAGCCGAAACTCAAGTACACCCGGATGACGCATGATCTCGCCAATATATTATTC
AAGGATGCTGCTAGCTGTGTTGCTCTCCATCCTAAATTCATCTGCGTTGGAACACGTTGGGG
CTCAGTGTACCTGCTGGATCACCAGGGTAACAAATCACAATCAGGCGTCACGCTTCGTTCT
CATTCAGTTTCTGTCAATCAAATTAGTATGGATGCCAAAGGCGAACATGTAGCTACTTGTTC
GGATGATGGTAAGGTATTTGTTTATGGACTTGATAATGTTGAGGATAGTTTCAACATGGAAG
TGCCTCGATTGGTGCGTTGCGTGGCTATCGACCCGCTCTACTACCAACCACGTTCAGGATCT
CGTTTTGTTACTGGAGATGAAAGGCTGGTTCTTCACGAGAAAGCATTCTTCTCGCGGTTCAA
ATCAACAATCCTGAGTGAAGCGAAGAGCGAGGGTGGTGTTCAAAACATTCGCTGGAGTCC
AAATGGCTTTCTCATTGCCTGGACCACCAATCTCGGCGTTAGAATCTACGACTTGAATGCTC
GATGTTCGCTTGGATTGATCAAATGGACAAGAACGTCAAACCTAGAACAGTACAGATGCA
ATTTGTGCTGGAAAGATGGACTAACGTTGCTGGTTGGATGGTTCGACACCATTCGAGTATGC
CGGGTCAGAAAGAAAACAATTCCAGAAATCTCTGAAACGAAAAACAGCTCAGAGTTCCTA
GTTGAGCCTGTGTGCACATTCCAGACGGAGTTTGTGATCTGTGGAGTGGCGCCGCTGGGCG
ACCAGCTGGTGGTGCTGGGCTGGTGCCAGGATGGGGGTGACGGGGAGGGGGGAGAGCCGC
AGCGACCGCAGTTGCACGTGATCGAGCCGCGTGCAGCCGACACCTTTCTCGACGCATCCAC
CGACAGTCTCACGCTGCGCGGGTACCAGCACTATTCTGCCAACGACTACCATCTAGAGTGC
CTTGTTGAAGAGAACCAGTTTGTGGTTGTCAGCCCGAAAGACATCATCCTAGCTAGTCCTTA
CAATGCTGATGACAGAGTCGACTGGCTGTTGGAACATTGTAAATTTGAAGCAGCAATGGAA
GCTGTTACAAGCAACAGTGGAAGCTTGATGCGACATACCATTCTGAGTGTTGGCCGTTCCT
ATCTGGACCATCTACTCTCGCTCAACAAATATCATCAAGCAGCTAAACTATGTGTTAAGAT
ATTAGGGAAAGATAAACGACTTTGGGAAGAGGAAGTATTCAAGTTTGCGCGAGTGCATCA
GTTGAGAACAATCAGTCCTTACCTTCCACAAGGACAAAACCACTTGGATCCTCATATCTAT
GAAATGGTCCTTTACGAATATTTAAAATTGGAACCAACGGGTTTCTTGAATGTTGTCAAGGA
ATGGTCTCCAAAGTTATACAATGTGCCTGCTGTGATCAATGCTGTGATGGAGATATTTTTAG
TTGGCGAAACTTCACATCGTGCTATATTTTTGGAAGCTTTAGCGATACTTTATTCACATGATG
GACAATATTCTAAATCGTTGGCTATGTACTTGAAATTGAAAAATATTGGAGTTTTCGATCTT
ATTCATCAGTATTCGCTTTACGATGTTATACATGACAAAATAGAAGGCCTGATGGAGTTGG
ATTCTTCACGAGCAGTTACGATGTTACTCGAAAAAGAACGAGTATCTCCCGATGTTGTAGT
AGCTCGTTTGCAGAACAATAAAATGTTCCTGTTTCTTTATTTGGATGCACTGGAGGAGAAGC
GCGACATGAAGGTGCAGTCGCGGAAGTACCACAGTATTCTGGTGAGGCTGTACGCGGACTT
TGCCCGCGAGAAGCTGTTACCTCTGCTCAGGCGCAGTGACCACTACCCCATCCAGGAGGCG
CTCAACATTTGCAAGCAGCGCCGCTACAACCCCGAAATGGTCTATCTACTAGGTCGTATTG
GAAATACCAAAGAAGCTCTCGATTTGATAATAAAGGAACTGAAGGATATCGAGCAAGCGA
TCAACTTCTGTAAAGAGCATGATGATGAGGACTTATGGAAGATCTTATCAATCATTCATTA
AAGGTTCCTGAGTACATAACATTCCTTCTGCAAAGGATCGGTACATATGTTGATCCAAGAA
TATTGATACAGAGGATACAGGACGGCTTTCAAATTCCCGGTTTGAAAAATTCGCTAGTAAA
AATAATTACCGATTACAATTTGCAGGTATCCGTCCAAGAAGGATGCAAGAAAATTGTGGTA
TCTGATTATTTTAATCTCCATAACAGATTGGTTTCAATGCAGCAACGAGGCACCTGTGTTGA
CGATGAAAAGTATTGTGGTGCTTGTCATCGAAAAATAATAATGAAAGATCTGAGCCAAGCT
GATGATATTCTAATGTTTTATTGCAATCACTCATTTCATAAACAATGTCTACCTGCACTTGGT
GTGGTGAGTTGTACCATATGCAGATCGCAGAGGTCTGATATTAGTCATCCAGCCATGACCTT
CGATTAG

Primers for *chr02.0345*.

Primer usage	Direction	Primer sequence (5′–3′)	RNAi efficiency (%)
qPCR	F	CACGAGCAGTTACGATGT	
	R	ACAGCCTCACCAGAATACT	
	F	T7-GCAAGCGATCAACTTCTG	88.24 ± 0.81
	R	T7-ACAACTCACCACACCAAG	

>chr03.1220

ATGATGACCCTCTCAGGACACCCACGCCCAGACACACCAAGGACAATCATGACTTCCCGTT
TTGCAAGATATTCTTCAAATATGGAAACAGTCGAGGCAACAATAGTTGAAATAGTCAAAG
AGCACCTGAACAGACGTCAAAGTGTGGAGAACATTTCAAGAAATTTTCTCAAGTTCCTGGC
ATCGACTTGTGGTCTTGTTGAAATACGCGTGCTGGTAGCACCTCGACTGGAGGTGTGGCTGC
AGAACCCAAAGCTGATGCGTGGCGCCCAGGAGCTGCTAATGTCTCTGTGCGTCAACAGCAC
GGCGCACACGCAGCGTGACGTCGAAGTCATCAGCGCACTCGTCAAGATACGTCTCAAGAC
AAAGGCGCTCATCAACTACTATCTGGCCTGCATCAAGGAACTGATCTCAGCTCACACGGAC
AATCTGGCAACAGTGTTGAAGCACACCATCTACAATGAGCTGTCCTCGTCGCACAACCCCA
ACAACATGCCCATGCTCGGAGTCATGCTGCAGTACAGTCCTGACCAATCTGCTTCTCTACTT
GCCGATATATTTCAGGAGCTGCTGCTGAACCGTGACGACTTCCTGCGCCCGCTGCGCGCGTT
GGTGCGCGAGGTGTGGCGTGTGTTGCGTGCGGACCTGCACTTGGCGGCGCTGTGCCGCGGC
CTCATGAGCCAGACCGAGCCTCCAGCCACGGCCGATGCGGCCGCCACGCAGCGCGCGTTC
AACTCCATCGCCGACCTCATCACACTCTGCATATTCCTCGCCGTCGCTCACCTCGCGCGCCA
GGACAAGAAGGATCCCGCTCAGATCGAGAAGATGCAGCTGACTGTGGCTGGCATTCAAAG
TGATGCGATTTGGTGGCTGCAAGAAACTGCTTTACGAGTCTATCGACCCAGTTCGTCGGATT
TTGTCCATGCTCTTCATAAGGTTTTGTTGATGGAGCCGGCCGAGCAGTACTACAAGACAGA
CAGCTGGCCACAGGAGCAGGACCGCGCGTTGTTCCTGCGGCTGGCATCCGAGGTGCCCGTG
GAGCAGGCGACGCTGCTGCGGGTTCTGCTCATTGGCCTCAGCAAGGAGCATCCGCTCATGC
CACCCGACGCACTCGAGCTCGCCGACCAGCTCGTCAAGCGTGCGGCCGCCGTCACTGCCAA
CGCCAACCTGCCCATGCTCAGAGCGGACAAAGTGGAGTTGATGGAGCTGGTGTTCCAGCTG
TGCGCCTACCACCACCCGGAGAACATAGACCTGCCAGCCGGGTATGCCCCACCCGACCTG
GCCATCACCAACCTCTACTGGAAGGGATGCGTCCTCCTGCTCATACTCGCCGCACACAATC
CACAAACTATAGGTGCGGTGGCGTGGCACAAGTACCCGACTCTTCGCGCGCTGATGGAGAT
GTGCATCACCAACCACTTTGTGTTTCCGGCCGGCGCAGCCGCAGATGACCTGCAGCTGGCT
GCACTCGAGAAGCAGACCATTCTGCAGTTCGAGACGCATCTCGCTGCTGCATCCACCAAGA
TGGCAATCACGCAGCAGACGAGCCTGCTGCTGTCTCAGCTGATGAGCATGGAGGTGGGGG
GTCCGGCGCGACAGCCGCCCGCCGCAGTGCTGGAGCAGCTGCAGTCGCTCAACCTGTCGCT
GCGACTCGGCCACCTGCTCTGTCGCTCGCGACACCCCGACTTCCTGCTCGACATCATACAGC
GACAGGGAGCCTCCCAGTCCATGCCCTGGCTCGCCGACCTCGTGCACAGCTCTGATGGATC
CCTCAATCACCTACCAGTGCAATGCTTGTGCGAGTTCCTGTTGAGCTCGAATGCGGTGGCCA
AACAGCAGGAGAAGTACCAGCAGCTGCTCACTCACCTGCAGTCCATACTCAGTGACCCCA
ACCAAGACCCGCTCATTGTCTGCGAAATCCTCGACTACTTCCTCCGAAGGCTGTCATCGCCT
CATAGTCGGGCTCAAGCTATCATGGGGATGAAATTAGTTTGAAAACGGTGAGCGACGAA
GAGGCAATGGAAACAGACAAGCCAATGGAAGAAGTGAAGGATGACGCCTCCTGGCTTCTC
AAGCAGCTGCCCATGTTGCCACATTTCAGTATTGCCAAGTCTCAGGTGGTGCTAGCACTTCG
CAGCGCCTGTCAGGTGGAGAACGAGCCGGCGCTGGTGACCGCCTACCTGTGCTTCCTGGCG
CAACACGCGACCGACCAAGGCAGCGCCGACATGGCAGACATGAGTGAGCTGGTGGTCGAC
ATGGCGCAACTCATAGTGGAGCGGAGCACCATCATGGCCGCCATCTTACCCACCCCCGCCA
CCGCACCCGCCTCCAACCCCTCGCTCGTCGCGCTCATCGCCATCTTCTCCAGCTACCTCGTC
AAGGCGCGAGAGCCGCGCCGCGAGGCGTTCACCTGGTCTGAGAGCCAGGACCAGATAGTG
GTCTCGTGGCCGGCAGGTGAGGACTGCACCCTGCACATCCTGGTGGTGCACGCCATGGTCA
TCCTGCTCACCTATGGCCCACAGGCGGTCGTCGAGCGCCTGCCCGCCTACAACTACCTGCTT
GACGCCTGGTTTCCACAGGACAAGGAGAGTGCTCCAAAGGCTTTCCTGGTGGATACATCTG
AAGAGGCTCTGCTGATTCCTGATTGGCTGAAGCTGCGCATGATCAGATCGCAGGTGCCGAG
GCTGGTGGAGGCGGCCTTGACTGATCTGGAGCCTCCGCAGCTCATTCTTTTCATTCAGTCGT
TTGGCATCCCTGTCGCATCTATGAGCAAACTGCTGGAAACGCTGGACCGCGCCGTGATGGG
GGCGGAGCGAGAGGCGGTGGGGGAGGCGGTGATGGACAAGTCGTACATGGTGCAGCTGGT
GGAGGTGCAGCACAAGAGGGGGGCCACAGGGGGGGAGGTGTTCGCCAGGGTGCTCGGAC
TGCACGAGCCAATCAGGGCAGCGCCCGAAACGCCCATCATTGCCGAGCCACAATTCCCTG
ATCTGAAGCCACCTGTTTGTATCAAATTGAAAGCCTTAACATTGCAAGAAGCAATCGCCGT
CTTTGAGCAAATCTATTCAACGCAAGAGGAAACTGAAGCCAAACGGCTCATCAATAGAGA

AGATTTCAAACTGTTACAAAGAACGCTGTCGGTGGAAGTAAGCAAGAAGAGCACCAGATC
AGGTGGTGGTGGTGGTGGTGGCCTCTGCCAGAACCTGCTAGTTCACCTACTGAAGCGCCTCT
CATCCGACCGCACACTATTGACTGTTGTCGCCGCACGCACGCACTACTCCTGCCCGCTGTTC
CGTCTGCTCACCTCGGTCACCTGCCAACAGCTGACTGCCGTCATGCTCGTCATCTGCAAGGT
GGTCGTCGACTCCCCCCACTGCTCCACCATGCTGGCCGCAATCTTGAAGGCGTTCACCAAG
CGCCACCAGTCCAGGGAGACCGCTGTCGTACCTGCCACCAGCGCATACCAAGCGCCGCTA
AAAGCTTTGGAACACTTTCCACGTGGAAAATTGGAGCGAGTGGGTCGCAAAGTGCTCGAGT
CGAATGTAGGCAAGGCAGTGATGGTGGAAGCAGTGGGGGACCTGATGCGATTGGTCCCTC
AGCGTCTGCCCTACTCCACCACCGGTCTGCTCATTGATTGGCTCATTCAAATCGAGCCCGAA
ATCATCAGCTCATCCACTGATTTCCAGATGAGAGCCCTGTTTTCAAGATTGGACAACAAAG
AGTTCATTTCGTTCAATACATGCCGACCTTATCTGTTGACCCTGCTCATTCATCAAGCCAGCT
GGTCGACGCTTCATCATTGTATGAATGGTCTTCTTCAAAATACTATGATTTCAAGGTATGAT
CCAACCGCTGTTCTAGATTTCTTGACTGCTTTGACTCGAAATCCGAAATTGTGGCAGGGAAG
GGACAAGTATCTGCCCAAGCACTACCAACCAGAAGATATACTGAATCTCTCTTCTGATCAG
ATAATTTACGTTGCGGATTACATTGTGGAGGAAGCAATTTTGATGGTTGATAAGAAGGAAA
GTCTCGATAAGATGAATCATCGAATTCAACTGCTCTCTCAGTGCATAGTAGAAGATGAAGT
TCATATGACAAGTTGCTTGGTCCGTCATCTTAATCGTGTTATCCACGATAGCACTGGCATTA
AGAATGAGATGTGTCGCCAACTTATGATGCTGCTCTACCTTCGGGTTCCCAGTCTTATTAGC
CAGGCCACCGATGTCAATCTTGATAGGTACTTGGACAGTACACTGGTATCCGGTAGTGTAA
ATAGTGTGCTAGACAGGATATCGCACACAATTGTGACGGCGTTGACAGCCACGCCACATGC
TAAGGACTGGGCCAGGCGATCGCAAGAACTCGAATTGGCCGCCAGAAAAATGGCCAGCAC
GCATCCTCTACTAGTTCTCAGGCAATTGAAAATGATTGGAGCCGCTCTGTGTGGCAGAGTGC
ATCTTGAATCAAGCGTGCTTCGATCGCGGGGGCACTTGTCTCTGTTTCAACATGTCTATGGG
CTGCTCGAACTGTTGAAGCCCAAAGTGTTTTCCGAAGACTACACGGAATCGCTGCAAAGTA
TATTGAAATTGTACTTTCAACTTTTTAAGACGCACGGACACATGAAAGAAGTCATCAACTTG
ATGAATAAAGTCGTCAATTTCCTACAAGATTACATCAGTCATGATGCCTCTCGCGCACTTTC
CTACCTTCAGAGCCAGTCAAACATTATAAGTGAGTTGCAGTCGCAACACTCGAACTTCAGT
GGCTTGTGCATCATGATGTCTGGGGTTACGGGGGGAGGGGAGGTTGTGGTGGCTGTGCCCC
CCTCCAATCAGCAGGCGGCTACGCCCCACTACGACTTGACCAATCTGCTGCGAGGCGAAG
ACGTGTTGTCGTGCCTGCAAGACCTTGACAAGCAGTCGGTATCGCGGCCTGCGCTGCTAGA
GCCGGCCCTCGGCTCCCTGTGTCGCTACCTCAGTTCGCGCAGCTCGGCCACGCGCAGTCTGG
CGCACGGTCTGCTCGCGCGCTACCTGCGCCACGCGCCACGTGCCGCGCGCGCCGCTCTGCC
CGCGTTCATCTCGTGCCTCGACTCGCCGCACCCGGAGGTCATCGCCTCTGCGCTCGACCGTC
TGCCCGAGATCACTGTCTGCTCGCAGGAACTGGCTTTACCGCTACTGAAGAAAGTGTTTGGT
GTAGGAATGAGCTCAAGCATAAATACGACGCAATGCATAACAAAGACTCTAGCACTGCTG
AATCTCCAGTCTGGATGCTAG

Primers for *chr03.1220*.

Primer usage	Direction	Primer sequence (5′–3′)	RNAi efficiency (%)
qPCR	F	ATGCCGACCTTATCTGTTG	
	R	CTGGTTGGTAGTGCTTGG	
	F	T7-GCTTGGTCCGTCATCTTA	98.32 ± 4.56
	R	T7-GCGATTCCGTGTAGTCTT	

>chr03.1450

ATGGGAAAAGCTAAGAAATCAAAAAAGCTAGGACGAGAAGATGATTTGGAAGATGATATT
GACATCAAAAGTGAAGATGGGGAGAAGAACATCAAACCCAATGAAAAAAAATCTTCCAA
ATCCAAAAGTAAACCGAAAGATGATGAAGATGTGATAGACAGCGATGCAGAGAATGAAA
TGGATGATAGCAAAAGCAAAAAGAAGAGCAAAAAGAGTCTGAAAAAGAAAAGTGTCGAG
GACGATGATGATGTTGATGAAATCACTAATGAAATATCAGAACTCTCAACAAATGATAGA
AAGAAGAAAAACAAGGGTAAGAAAAGTGTTGCTGACAACTCAGATAGTGAAGAAGAGGC
TGTGAAAAAGCCAGCTGCAAAAGGCAAAGCAAAGAAGAGTGCCGGCTTTGCTCTGTTAGC

TGTTTCGGACGACGACGACGATGATGATGATGAAACAGCTGTCGCGCCTGATCCACCTTCA
GAGGACGAGGCAGCTGCAGCCGAAGCGGAGAAGAAGAAGGAGAAGGGAGGCAAGAAGG
GCAAAAAGGGAAAGAAGAAGAAGGATGAAGACGACGAAGAGGATTTGGACAAAATGCT
GGCCGAGTTGCAAATGGAGTATGCCGGAGTGAAGGAGCCCAGTGTGGAGAAGACCGCCGC
CGACGAGGAGCCAGCAGCCGCACAAGTGGACGAACCCACCAAGTCCAAGAAAAAGAAGA
AGAAGGGAAAGGAGGAGAATGAGAAGGTTTCTGAGGAGAAAGGTGAAGATGATGATGGT
GACGAAACAGCCGGCGGCACTGTAAAAACTGCTGCGCAAAAGAAAAAGGAGAAGAAGGA
GCGCGAGAAACAGAAGAAAATGGCTGCCAAGAAACAAGACGTGGAAAAGAAACCTCAA
GATCAGAATGAACCAGAGACTAATGAACCAGATGCCTTAAAGGAAGGAGACAACGCACA
AGAAGAAGATGGCGATGATGATGACAAAAAGAAGAAAAAGAAAAAATCGAAAAAAGAG
GAGGAAAAGGATAAGAAAGAGAAGAAGGGTCCAGGTAAAAAGACAATAGCCGCAATGC
AAGATGCACTGAAGAAATTGCGAGAGGAAGAAGAACGTGCAAAGAAAGAAGAGGAAGA
GAAATTGAGACTAGAGGAAGAGATGGAGATGAAGAGATTGGAACAGGCAAGACTAGAGC
AGGAGAAGAAAGAGAAGAAGAAGCAGAAAGAGAAGGAGAGAAAGGAAAGACTGAAGG
CGGAGGGGAAGCTGTTGTCGGCAAAGCAGAAGGCGGACCGAGCGAGGGCGCAGGCGATG
ATCGAGGCACTGCGAGCCAAGGGCCTCGACGTCCCCACGCCGGGCGAGAAGAAGCCTCCG
CGCCCGGGCACGCGCATCCGTCCCAAGAAGAACAAGAACGAACAGGCCAGCGACACCGT
TGAAGAAACAAAAGATGAAGAAGTACAAGAGCAAACTGTTCAGGTGGAAATAGTTGACA
AAAAGGAAGTACTCAAAGTTGAAGAGGATGTAAAGGACTCGTGGGATGCTGAGTCATCAG
AAGATGAAGAGAAATCCGAAGAAACAGAGAAACCAGATGGTGATAAGAAGAAGAAAGA
GGCTGAAGATGACGAAGAGGAGGAATCGTCGAGCGAAGAGGGAGAGGGAGAGACAAGC
AGTGAAGAGGAGGACGAAGATGATGAGTCAGAGGAAGAGTCGAGCGAAGACAGCGATGA
CGGCGAAAAGAAGACTGACGCTGAAAGGAGAAGAGAGAAAGCCATTGCAAGAATACAGA
AAAGACGAGAAGATGCTGAGAAGAATAAGAGCTTAAATGAGCTGAGAGCTGCTGTTGTAT
GTGTTCTGGGACATGTAGATACGGGTAAGACTAAGATTTTAGATAAATTACGAAGAACAAA
TGTGCAAGATGGTGAAGCTGGCGGTATTACTCAACAAATTGGAGCAACAAATGTTCCATTA
GAAGCTATCAAAGAGCAAACAAAAATAGTCAAAGGATTTTCAGAGATGGAATTGAAAATC
CCTGGACTGCTAATTATAGATACACCCGGCCACGAATCGTTCAGCAATCTTAGAAGCCGTG
GCTCCTCCCTCTGTGATATTGCGATTCTCGTTGTAGATATTATGCACGGACTCGAACCTCAG
ACCATTGAGTCGATTACTCTACTCAAAAACAAGAAAACACCATTTATTGTAGCTTTAAATA
AAATAGATAGGTTGTATGATTGGAGTAGTATGGCTAGAAAAGATGTAAGAGATGTCATAA
AAAGTCAGCCTTCAAATACTCAATTGGAATTTGAACAACGAACTAAAGAAGTTATTATTCA
ATTCAATGAACAAGGTTTGAATGCAGCTTTGTTCTACGAAAATCCGGACCAGCGCAGTTAT
GTCTCACTAGTTCCCACATCGGCTATCACCGGCGAAGGCATGGGAAATCTGTTGGCGCTTAT
AATGGAATCTTGTCAAACTATGTTAGCAAAAAGACTCATGTTCTCCGAAGAATTGCAGGCA
ACAGTATTAGAAGTGAAAGCCATCCCAGGTCTGGGTACGACGATAGACGCAATCCTGGTG
AACGGAAAGCTGAGAGAAGGAGACACAATGATAGTGGCCGGCACGGACGGACCAATCGT
GACGCAGATTCGATCGCTGCTGATGCCGCAGCCAATGAAGGAGCTGCGAGTGAAGAACGC
CTACCAGGAGTACAAGGAGATCCAGGCAGCGCAGGGAGTCAAAATTGCCGCCAAAGACTT
GGAAAAAGCCATAGCTGGCTTGAATCTGTTGGTTGCACAAAAGCCGGACGAGGTGGAGGT
GATGAAGGAAGAGGTGGCCAAGGAGCTGAAGAGTGCTCTGAGCTGCATCAAACTGGCCGA
ACGGGGTGTCTACGTGCAGGCGTCCACTCTTGGCTCTCTCGAGGCGCTGCTCGAGTTTTTGC
GCACCTCCAAGATTCCATACTCGAACATCCGCATTGGTCCAGTTGTGAAGAAGGATGTTAT
GAAAGCATCAACTATGTTGGAGCACGACAGTCAGTATGCTACGATTCTTGCATTCGATGTG
AAAATAGAAAGAGATGCACAAGAGCTGGCAGATTCGGTTGGAGTGAAGATATTCCAAGCC
GACATAATTTATCACTTGTTCGACAAGTTCATGGCCTACAGAGAACAGCTCAAGCAGAAGA
AGCGCGATGAGTTCAAGCATGTTGCCGTGTTCCCATGCAAGCTCAGAGTGCTGCCCCAGTT
CGTTTTCAATTCTCGTGACCCAATCGTGATGGGTGTCATCGTAGAAGCTGGAATCGTGAAGG
AAGGCACGCCGATTTGTGTTCCTAGTAAAGAGTTTGTGGAGCTGGGTATTGTGACAAGTAT
AGAAAACAACCACAAGCCGGTGGAGTCGGCGAGAAAAGGCCAGGAAGTGTGCATCAAGA
TCGACCCGATTGCCGGCGAATCGCCCAAAATGTTCGGCAGGCATTTCGACGAAAAGGACA

TGCTTATCAGCAAGATAAGCAGACAGTCAATAGATGCTTGCAAAGATTATTTCAGAGATGA
TCTTATCAAGGCAGATTGGCAGCTAATGGTCGAACTGAAGAAACTTTTCGAGATTCTATAG

Primers for *chr03.1450*.

Primer usage	Direction	Primer sequence (5′–3′)	RNAi efficiency (%)
qPCR	F	AAGCCGGACGAGGTGGAGGT	
	R	AATGCGGATGTTCGAGTATGGA	
	F	T7-GGAGAAGAAGAAGGAGAAGG	96.6 ± 3.82
	R	T7-GTCATCATCATCGCCATCT	

>chr04.0290

ATGGACGCCTTTCTAAGCAATGGAAAGGCCGCTGGCGGCGACGCTGGCAATAAGACGCCG
ACAGGCAAAATTGAGAAGCAGTATCAGAAGAAATCACAGCTTGAGCACATTTTGCTCAGG
CCTGATACATATATTGGTTCGGTTGAACACCACACGGAGCAGATGTGGGTCTATGACTCGG
AGAAGAATGCGATGGAGAAGCGTGAGATCACCTACGTTCCCGGATTGTTCAAAATTTTTGA
TGAAATTCTCGTAAACGCTGCCGACAACAAGCAGCGAGACCCCACAATGAACACTATCAA
AATTGAAATTAAACCGGAAGAAAATGTAATTTCGATCTACAACAACGGCAAGGGCATTCC
GGTGGTGCTGCACAAGGAGCAGAAGATGTATGTGCCGACGATGATCTTCGGCCACCTGCTG
ACCAGCTCCAATTACAACGACGAAGACGACAAGGTGACCGGTGGTCGCAACGGCTACGGA
GCCAAACTGTGCAATATTTTCAGCTCGTCGTTCATCGTCGAAACCAGCTCCAAGGAGTACA
AGAAGAGCTTTCGACAGAAATGGGCCGACAACATGTCGAAAACTTCGGAGCCGAAAATCA
AGGAAGATGACGGAGATTTCACGAAAGTGACATTTTCGCCGGATCTGTCCAAATTTAAGAT
GGATAAGTTGGACAAAGACATAGTCGATCTGATGTCAAGGAGAGCCTGCGACGTTGCCGC
GTCGACTGGCGGCGTTAAAGTGTTTCTCAACGGCAAAAATCTTCAGATTAAAACGTTCAAA
GATTATGTTGATCTATATACGAAGGATAAATTAGATGATGATGAGAATCCTCTGAAGATAG
TCCACGAGAGATTTGGCGCCCGCTGGGAAGTAGCCGTCACATTATCCGACCAAGGATTCCA
GCAGATCTCCTTCGTGAATAGCATAGCCACAACAAAGGGTGGCCGCCACGTGGACTATGTA
GTGAACATGATCGTTAAGAACATAGTGGCAACAATGAAGAAGAAAAACAAGGGCGGCAT
GGACATGAAGCCGTTCCAGATCAAGAACCATCTGTGGGTGTTCATCAACTGTCTCATCGTC
AACCCGACGTTCGACTCTCAAACTAAAGAGAACATGACGTCACAGGAGAAGAACTTCGGC
TCCAAGTGTGTGCTCACCGACAAGTTCATTGCGGGCGTGCTTAAATGTGGCATTGTCGAAAC
GCTACTGGTCTGGGCCAAAACCAAGGAGCAGGAGAAACTGCAGAAGGCGTCGGGCAAGA
AGCAGACGCGGCTGAAGGGCATTCCGAAGCTGGAGGATGCCAACGACGCCGGCAAGAAG
GACTCTGTCCACTGCACGCTGATCCTGACCGAGGGAGATTCGGCCAAGACGCTGGCTGTGT
CCGGGCTGGGCGTTATCGGCCGTGATCGCTACGGCGTCTTTCCGCTCAGGGGTAAAGTGCTC
AACGTCAGAGAGGCCAGTCACAAGCAGATTTTAGAAAATGCTGAAATAAACGCAATAATA
AAAATTACTGGTTTGCAACACAAGAAGAAGTATGAAACTATTGAAGACATGAAAACGTTG
AGATATGGAAAAATAATGATCATGACTGATCAGGATCAGGACGGTTCTCACATTAAAGGTT
TGCTAATCAACTTCCTGCATTTCTATTGGCCGTCTCTGCTGAAAATGAATTTCATCGAACAGT
TCATCACCCCGTTGATAAAAGCGACAAAGAAGCAGAATGTTCTCTCGTTCTTTTCGATTCCC
GAATTTGAGGAGTGGAAGCGAGGAACGGATGACTGGCACACCTACAAGATCAAATACTAC
AAAGGTTTGGGAACCTCCGATTCAAAGGAGGCTAAAGAATATTTCTCGAACATGGAAAGG
CACAAAATCAAATTCAAGTACACTGGAGAGGAATGTGACAAAGCTATTCAGATGGCGTTC
AGTAAGAAACAAGTGGAAGCGAGAAAAGAATGGCTGACCAACTGGATGGTGGAAAGCAA
ACGCCGACAAGAACTCGGTTTGCCCGAAGAGTATCTGTACGGCAAAGATACGAAGGTCAT
CACCTACAAAGAGTTCATCAACAAGGAGCTCATCCTCTTCAGTCACATGGACATTGAACGG
TCCATTCCCAGTGCTGTCGATGGCCTCAAACCTGGCTCAAGAAAGGTGCTGTTCACATGCCT
GAAGCGCAACGACAAGCGTGAGATCAAGGTGGCCCAGCTGGCCGGGTCGGTGGCCGAGC
AGTCGGCCTACCACCACGGTGAGGTGTCACTGATGTCGACCATCATCAACCTGGCGCAAAA
CTACGTGGGCAGCAACAACATCAACCTGTTGCAGCCGCTCGGTCAGTTCGGCACGCGACTG
CAGGGCGGCAAGGACGCCGCAAGTCCGCGTTACATCTTCACCATGCTCAATCCGCTCACGC
GACTCATCTTCCACCCGGACGACGATCCGCTGCTCAAGTACCTCAAGGACGACAACCTCAG

GGTCGAGCCGCAATGGTATATGCCGGTGATACCGATGCTGCTAGTGAACGGCGCCGAAGG
CATAGGTACTGGCTGGATGACGAAGATCCCGAACCACAATCCGCGCGAAATCGTTGCCAA
CATCAAGCGCATGATGCAGGGCGAGGAGCCGCTGCCGATGAAGCCGTGGTACAAGAACTT
TCTGGGCGAAATCGATCCGCTCGGCTTGGAGAAGTTTGTGGTGAACGGCGAGGTGGGCATC
ATCAGCGACACTCGGCTTGAGATCACAGAGCTGCCGGTCGGCACCTGGACGCAGAACTAC
AAGGAGTCGGTGCTCGAGCCGATGCTGCATGGTGCCGACAAGACGCCAGCTTGTATCACTG
AATACAAGGAGTACAACACAGACGCCACAGTAAAGTTTGTCATCACAATGTCAGCTGATA
AGCTCGCGCAAGCTGAAAATGAAGGCCTGCACAAGTTTTTCAAGCTGCAAACACTCATCAA
CATCACATCTATGTGTGCGTTTGACAAAGACAACTGTTTGAAGAGATACACCTCGGCGATT
GAAGTGCTGAAGGAATTTTTCCCCGCAAGGATGTTGTACTACGAGAAGAGGAAAGACAAT
ATGGCCGGCACTCTCGAAGCCGAAGCTAGAAAATTGTCCAACCAGGCAAGGTTCATCTGTG
AGAAGTGCGATGGCACACTGGTCATTGAGAACAAGAAGAAGAAGGATATGGTGGCCGAAT
TGATTCGCAGGAAATTCGACCCGGATCCAGTCAAAGAATGGAAGATGAAGAAAGACAGA
GAAGCTGCTCTGATGGATGATGATGAGGAGGCAGCAGCGGCTCCCGGCTTGTCGCAGACTG
ATGATGATGGCGGTGAAGGGGATGAAGAAGGGGAGGAGCGACAGAACACCCTGGACAGT
AACTTCGACTACCTGCTCGGCATGGCAATGTGGAACCTCACCAAGGAGAAGAAGGATGCG
CTCATTCAGAAGAAGGACGAGAAGCAGCAGGAGTACCGTATCCTGCTGGCAAAGACCCCG
CAGGACCTGTGGGTGGCCGATCTCGATACGCTCATCGAAAAGCTGGACGAATACGAGGCA
AAGGAGCGTGCCGAAATGCTGGACATCAAGGTGGAGGGCGGAAAGGGTGCAAAGGGTGG
GGCCAAGAAGGGCGGGGCCAGGAACAAGAACAAGGTGTTGGTCACTGACTACCTGCCGTC
GCCGATCGCCCGCCGTGTCGCGCCAGTCATCTCCGACGAGTTCAAGAAGAAGGTGGAGAA
AGCAGCTGCTGCTAAAGAGAACAAGGGCAAATCCAAAGCGAGAAAGAAGTTGGGAGACG
AGGAGGTGGACGAATTCGACTTGATGAGTGAGAAGAACCAGGTGTCACTGTCGGATCGAA
TAGCCAACACGCCGGCGAAGAAGGCTGCCGAGAAGAAGCCACGTGCTAAGAAGGCCGAG
AACGGCGAGAAGAAGCCGAGGAAGCCGCGAGGCACCAAGGGCAGTCCGAAGAAGAAGA
GCAAAGCGGCGTGGGAAAGTAGTAGTGAGGATGGGGCAACAGCGATGACTCTGATGCTAG
CGAGGTGCTGCCTGTGTCACCTGTACGCCAGACGGCTAGGAGATCAGCTGCCCCAAAAAA
ATACAATTTTGATGATGACGCTGACGATGATGATGATGAGGATGTGCTCTATGACAACGAT
GCTGCCATCAACGGTGATGAGGATGCGCCAGTCGTCGAAAATGATCTCGATGACAATGCA
AGCCTTAACGGTAACTCGTCTGGCAACGAGTCGATAA

Primers for *chr04.0*290.

Primer usage	Direction	Primer sequence (5′–3′)	RNAi efficiency (%)
qPCR	F	AACAAGTGGAAGCGAGAA	
	R	ATGTGACTGAAGAGGATGAG	
	F	T7-GGCAGCAACAACATCAAC	85.87 ± 1.04
	R	T7-GCACCGACTCCTTGTAGTT	

>chr08.0761

ATGGAACCAAACCAAGAAGTTGCGGAGTTCCAATCCTCTCTTCGTCAGTGGGAGGAGGATC
ACCAAGGCACCACCTATGATCCAGTTCCTATGCTGACAAGAATGGCAGAGTTAATTGAGCT
AGAATCTGAACATTACATGAAAAAAGATCCAGATCCATTCGACGAAAGGCATCCGTCAAG
AGTTGATCCAACCTGTGCTTTGGGACAGATGCTCAAAGTCTTGTTCAGAAAAGATAATTTCA
TGACTAAGCTTGTTAATGATTATCTGCGTGACAACTACTGGACACGACTTGGCCTAAATGA
GAGGGATGTTAGGAAGTTGAATGTTGCAGCTTGCAGGCTAATATTGGACATAATGCCTGGT
CTTGAAACATCTGCTGTATTTGAAACGCCCACAAATGATGCCTTGGTGAATCGGCTATACTC
ATGGGCTACAAGCTCTTCCGAGCCCCTCCAGACTTACGCCACAGGGCTACTCGCTGCAGCC
ATGGAAGTCCAAGATATTGCTGCTGCATTCAGGGAACAGAACGGCAAACTGGTCCCTCTAA
TGCTGGAGCGTCTGCACAGACTGCAGAAAGAAGCGATGCAAGAAAAGTATCCGATGGTGT
CTCCCAGCACTGGCACAAATCGACCTTTCGCCAACCTCGGACAAATGGACAGAGACAAAG
GCGGCGACAGTACACCCTGTCATAGGAAAGGCCGTGGAAAACAAAAACCGAACAATCAA
GAATCGCACACCTATGAGACCACTGATGACCCAATGGAAAATGGTAGTGACGCACCAGAA

CTTAGTAGCACTGTTATGGTCACTCCGCCCAAAATCAATGGATCAGTGAGAAGGAGCTCTG
ATTCTGAATCGTTGGTGGGCAGACGCAAAAGCATGGACTGCGGCATGTCGAGTCCGCCGAC
GCATGCGCAGCTGCGACCGTCTGCCGCGCATGTGCACGCGTCGCCGTTCAACGTGTCCGAC
TGCAGCAACTCGTCGTGGGTCGAGATGGAGTCCTACGTCATCGGAAACGTCCAAATGTTCC
CACCCATCCTCGTCACACGACAGATATTCATCCTCAAGTACCTAACGCCCATGGGTGAATA
CCAAGAGTTTCTTAGTCACGTGTTCGAAAAAAACGCTTTAGAATTAATCCTACAATTTATCA
ATCTTAGAGAATCGAAACATGCAAGACTGGCGTTTGAAGCATTGAAATATCTAGCATCATT
ATTGTACCACAAAAAGTTCTCCATTGAATTCATCAACATGAATGGACTACAGAAACTGTTG
GAAGTGCCAAGACCTAGTATAGCATCCACTGGTGTGTCAATATGTCTTTATTACCTTGCCTA
TTGTGAAGATGCAATGGAGAGAGTATGCCTTTTACCTCAGCACATACTTTCTGATTTAGTTA
GATATGCTTTATGGTTGTTGGAATGCTCTCATGACTCCGGGAGATGTCACGCAACCATGTTC
TTTGGGGTGTCATTCCAATTTAGAATAATATTGGAGGAATTTGATGCTCAAGATGGCCTTCG
GAAGCTTTATAATCTGATGAGCATGCTGCCGATTCTGTCGCCCGACGACCGCAACTATGTGC
TGAATGAGGACGAGGACTGCGCGGCGCGCCAGATTGTGCGTCACGTGTGCGTCGCACTCAA
GCGCTACCTGGAGGCGCACCTCTATGTGCGCGCCGAACAGCTGCGCCGTGCGCACATGCGC
GAATCCAACTCCGAGCACCACAAGATGCAGAGCACACTGCCCAGTTACAAGGCTGTTCGT
AGTACGCCGGAGGAAGTGAGAGAGCAGATAAGTGTGCTGCTGGAGCTGATGCCTTTCCGC
GCGCACTGGCAGCCGGTCGACCAACTGCTGCGACTCGGAGGCATCTCACTCCTCCTCCAGG
TCATCACCTTCTCCTACGACTGGAACTTCGCTGGAAGAATGGAAACAGTGCGCAGTGCGTT
GGATGCGCTAAGCATATGCGCAGTGATGCCGAACGTGCAGCTGGCGTTCTGCGAGCGACTC
GAGTCGCCCGACCAAACCAACAAGGTCGGACTCAACGTCATTCTGAACGCCGCCGAGGGA
GAGATCGTCGTCGACCCCGACGTGCAGAGGGCAGCGCTCGGTGTCCTCATAACCTGTGTCT
GTGCTCCCGTCTACAGGGGTGGAGGATGTATTGCAAGATACTCAGCGAGCGGATCTGCCAA
AAAGAAGACCCAGAATATGAAAAATAGTGAAGAAATTATTAATAAAGTATGGGAAGTTGT
ACGGTCGAATAACGGAATAATGGTGCTACTGCAGTTGATGATGGTGAAGATTCCACTGACA
GACGCAGACTCGATCAGGAGCATGGCTTGCGAGGCACTGTGTGGTCTCGCCCGGAGTGAG
ACCGTCCGCCAAATCATCAGCAAACTGCCCATTCTAACAAATGGACATTTGCAAAATTTAA
TGAGAGATCCTATTCTACAAGATAAAAGACAGGAGCATGTGGCATTCCAAAAACATGCCC
TGAAACTTTTAGAACGTGTTTATGGCAAAAGTAATGGAAATGACCTTGAAGTATCTCTTGCC
AATATTCACAGGGCGAACATCATAGCGCAAACGCGGATCCAGTTCAACGACAAACAGCTG
TTTCAGCTGTTGCAACAGCACCTGATCTCGCACGGCATGCTGGAGTCAGCTGCCACACTGC
AGAGGGAGGCGGGGCTGCCTCCGCCCCCCGCCCCCAAGCCTCCCCTGCCCGCCACATTCCT
GTCACCCTTCACCTATCAACAGCGCAGTCCAGGCCAGCCGCAGAGGTCAAGGTCTTCATTA
TCGCGGGAGAATCCACTACATAGAATAGCGCCATCTACTCAGACGTCGGATCTTGCGTCGC
CCTCCACCAGTCCACTGCGAGCCGGGTTCAGTGTTAGGGGAACCGCCTCTCCCTCCTCCTCC
TCCACCTCAATTTCCTCCCCCGCCAATCAGCCCATCAAGTTGACCATTGTCAACAACAAAC
GCAGTGAAATGAAGTTGACAGCAGCGCAAGCGCAGTCGAGCTCGAGGTCGCTTGAGAAGC
AGATGAGATGCGAGCCAGGCCAGCAGCCCGGGCATACGTCACTGGCGCCGCAACACGTCA
CGCTCGACTCCATCATCACCGAGTACCTCACCAATCAGCATGCGCTCTGCAAGAATCCCAT
TGTCACATGTCCGCAGTTCAATCTGTTCGAGCCTCACAAATGTCCAGACCCGAGTGCAAGG
AACAGTGCTCCTGTCAACTTTGCAGTGAGGTGGTCGAAAGGCATCAATTCCGACCGCCTCA
ATCAGAGGCTTGTCCACTCAAGGTTCTGTCCGGCAAAAACCTTCAGGCTGGTAGACGAAGA
TGGATTTTTTACCTGCTGCGAGTTCCTTGCAAAAGATAAACACGTTTTAATTGGAACACATC
AGGGAGATGTGAAACTATACAACACCCTTACTGGAAATGAAGAGGCAACTTACCAGTGTC
ACGAGTCTTACATTTCCAATATTCAGTCCAATAGAGATAGTTCTTTGATTTTGACGTCATCA
ACATTTTGGCCATTATCAGCATTATGGAAAGTTGATAATGGATTTATAAAGCAGTTTGCCAT
GGATGATGATGATTACGTCGAATTTAGTAAATATAGGCAGGATAAAATAATAGGAACTAA
AAGTGAAACTGCTACGATCTACGACATAACGACGGGCAGGAAGATAATGACGCTGCAGCC
GAGCAAGTCGAACGAGTATGACAAGAACAGAGCCACGTTCAGCCCGACCGACGAGCTGGT
TCTCTCCGACGGAGTGCTGTGGGACATCAACTCGGGCAAGGAGATCCACAAACTCGACAA
GCTCAATCAGACGCTTAGCGGGGTCTTCCATCCCAATGGACTCGAGGTTGTGTCGAACACA
GAGGTGTGGGACCTGAGGACGTTCCATTTGCTGAGAACAGTCTCCTCGTTGGACCACTGCG

AGGTGATATTCTCACCAACTGGCACGGCCATCTATGCCGTCTCACTCGAACAGGAGACCGA
CGAAGAATCCAACTACGAGTCCTCTTTCAAGACCCTCGATGCCCGCGATTACTCCAGCATA
GCTACAATCGATGTTAAGAAGAGCATCTACGATTTAGCAGTCAATCGGCTCGACACTCAGA
TCTTAATTGTGGAGAATCAAGGCATGTTTGAGAATGTGCAGGAATCGACTGTGAGACTTTAT
GATGTCGGCCGCAGAAGGGTCGATGAAGATGAAGTGGATGATGAAGAAGAAGAGGAAGA
ACTTGATGGAAGCGATGATAATTCTACACCCGATTCTCCTTCTGAAGATGAACAGGGAGAT
GTGTCAGCCGGTGACCTAATGGAATTTGTTGATGGCGATGATGACGACGACGATGATGATG
ATGATGATGACGACGATGATGGGGATGGAGATGACGATGATGGTGATGGAGATGATAATG
ATTTCGTATTAGTTGATGATGATGCTAATCCTGCGTCGCCTGATGCCGCAAGTGACGATGAT
GATGATGATGACGATGACGACACGTTCATTGTAAACGTCTAG

Primers for *chr08.0761*.

Primer usage	Direction	Primer sequence (5′–3′)	RNAi efficiency (%)
qPCR	F	TCATCACCTTCTCCTACGA	
	R	TTGAGTCCGACCTTGTTG	
	F	T7-GCTCGGTGTCCTCATAAC	90.32 ± 6.64
	R	T7-GGCAAGAGATACTTCAAGGT	

>chr08.1144

ATGGGTAAGGTTTCAACCCCCAAGTCCGGACAAAGTGGTGTAATAATTTCCAACGGTAGTA
AAGCAAAGACCCCTGATTTCAAAACAAAAATTTTAAAGAAAAAATCTTTTTATTTTGCTGAT
CCTTTCATTGTACCAAGAATAGAAACGTACCTTGCTGAAACAAGTGATGAATTGGAAGTTG
ATATTGATACTTTGATTGAACACCTCAGAACACAATACAAGGAGTATGGAAAAAGGAAAT
TTGGCCCTTTTGCAGCAAATGTCAAAAAAGCTGTGAAGTTTATAATGCAAAAGCACAGTGT
AGAATCAAGACCAGCTGAGGATGAGGATGAGGTTTGTGCAATACAGGATCAAGAGGAAG
ATGAAGAAGCAGAAGACTTCAACGTGGGTGAAATCGATGAAGAAGAAGAAGAAGAAGAG
GAGGAAGAGGAAGAAGACGAAGAGGATCTAGGTGTGTTGGATGATGATGATGATGATGAT
GATAGTGATGAGTTCGACGGGGACCCTCTTTCGGATCGCAACGGAAGAAGTGTCAATACTA
GCAGCAACAATACCATGATGGGAACCGAGAGAATCGGCAATGTCATTATCAATAGAGGTT
CTGTCTCAATGATCAAGAAAGACAATGACGTCAATAATACTTCTCAGGAGCTTATAAATAT
AAGCAGCGATGATGATGAAGATGAAGATGAAAAGTCTCCATCGCCTCCAAAGCCACCACA
AATTACATTCCAAAATGGAAAAGTGAATACAGGAACTAATTATTTGGTGAAAAAACAAAT
CCCCGGCCAACGGCCAATGATTAATAGCACCTATGATCCGAGTAAGGTGATGGGAGACAG
GCCTCAAGGTCAGCCTACTCCTGGGCCAAGAACCTTCACCAAACTTAGATTCGCCGAAGTA
AAGAATCCCAGTATCACGCTGAAAGATGTCGGCGGCTATAGCAAAGTGTTGAAGGATGTA
GTGGAGTTGGTGGTGCATCTGAAACATCCAAAGATCTACCACTTCTTGGGGGTTACTCCTCC
TAGAGGTTTCCTGTTACATGGTCCCTCAGGTACTGGCAAAACTCTCCTGGTTCGTGCTCTAG
CTGGGGAGCTAGGCGTCCCTTTGATCCGCGTTTCGGCGCCCGAACTGGTATCGGGAGTGTC
GGGCGGATCGGAGGAGCGGATCCGTGAGTTGTTCGAGCAAGGCAAGCGTGTGGCGCCGTG
CATTGTGTTCCTTGACGAGGTGGACGCCATCACGCCGAGACGTGAGAACACTCAGAAGGA
CATGGAGCGACGCATTGTGGCGCAGTTGATCACATCGCTTGACGAACTGTCCGAATCGCCG
GCAGGCAAGGAGATACTAGTGGTGGGCGCTACGAATCGTCCGGACGCCATGGAGCCGGCA
CTGCGGCGAGCGGGCCGCTTCGACCGCGAGATCTGCCTGGGCATTCCGGACCGCGCCGCCA
GGAGGGAGATCATCGCACTGCTCAGTCGGCAGCTGAGTCTGGCCGCCGATGTCAGTCTGGA
TCGCATCGCTATGGCCACGCCCGGCTATGTGGGCGCCGATCTCAAGGCGTTGCTCACCGAG
GCTGGTATTGCCGCTGTTACCAGAATTGTCGACAAGAAAATAAAAGAAACAGTGGAACTG
GAGCGAGCCAAGGAAACGGCGGACGTGCCAATGGTGGAGACCGCTGCAGAGGGGGCAGA
AGGAGGGGGAGAGCCGGCCGCCAAAAAGATGAAAACGGGGGACGGTGGTGGAGAGAAG
GATGTGTTGGAGAAGGGGGTGGTGGAAGAGGTAGTGGTGGGAGGGGAAGCAGCGAAAGA
TGAGGGGAAAACTGGTGGGGAGAACACGGCTAGTTCTAGTGGCAAGACTGCTGAGAAGAA
GTTGGAACAACAGGAGGAGCCAGATTTCCTTAACTACAAATGGCAGCTGTCGCGACAAGA
GCTGACCGCACTGAGCATAGAGAACGCTGACTTCGAGGTGGCCATGAAGAAAGTGCAGCC

GTCAGCGAAACGCGAAGGCTTTGCGACTGTGCCTGACGTCACGTGGGACGATGTCGGGTCA
TTGCAAGACATCAGGCAGGAGCTCAAGTTAACTATTCTGGGTCCAGTGCAGTTCCCCGGGG
CGTTTGCGTCGCTGGGGTTGAGCGCTGCGAGTGGGGTACTGCTGTGTGGTCCACCCGGTTGC
GGCAAGACCCTCCTCGCCAAGGCCATTGCCAACGAGGCTGGCATCAACTTCATCTCGGTCA
AGGGGCCCGAACTACTAAATATGTATGTGGGTGAGAGTGAGAGAGCCGTGCGACAGTGCT
TTGTCCGTGCCCGCATCTCGCAGCCCTGTGTCATATTCTTCGATGAAATCGACGCACTTTGTC
CAAAGAGATCCGACTTCGGAGATGGTGGCGCGAGTGCCCGTGTGGTGAACCAGCTGCTAA
CGGAGATGGATGGCGTGGAGGGGCGGACGGGGGTGTTTGTGATGGCCGCCTCCAACCGAC
CCGACATCCTGGACCCGGCAGTTCTGCGACCTGGACGCATTGACAAGATACTCTACGTTGG
CCTTCCCAACGCTGCCGACCGTGTAGATATATTGCGCGCGCTCACTAAGAACGGGACACAA
CCGACGCTATCGGCCGACGTGGATTTGGCCGCCTTGGGCGCAAGTGAGGCGTGCGCCGGCT
ACACGGGCGCCGACCTGGGCGCACTGGTGCGCGAGGCGTCCATCGAGGCGCTAAAGGAGA
TGATAGGGGCGCATGCACCCAGCAGTGCTGCCGCAGCCCAGTCACAATCCTCTATAAGTGT
AGCCGCAAGACATTTCACCGCTGCCCTCGCCAAAGTCAAGCCGTCTGTATCGGAAATGGAT
CAAAAGCGGTACGCAGAACTGAAATTGAAATACTCAGCTACTCCAGCTGAACAAACTATG
GAAATATCCGAATAA

Primers for *chr08.1144*.

Primer usage	Direction	Primer sequence (5′–3′)	RNAi efficiency (%)
qPCR	F	GCCAAGAACCTTCACCAA	
	R	ACCAACTCCACTACATCCT	
	F	T7-CTGGTATTGCCGCTGTTA	89.98 ± 4.91
	R	T7-CTCCTGCCTGATGTCTTG	

>chr11.0845

ATGGATGATCAAAATGATGGTTCGAAAAAGTCTCACAAGAAACCTCATTCAGGTCGTAAA
GCTGAGAAAAAGGATAAGAAGAAAAATCAACATGTGCAGGAATTGACGGACGCTCAGCG
AAATCCAAAAGCATTCACGTTCAATTCTGCAGTCCGAGCTGAGAGAAATTTTAGAAGAGGC
CAAGACATCCAGACGAAAAAGCACCATGTGCCGAAAGTAGACCGCACCCCGATCGAGCCC
CCGCCCATACTGGTGGTGGTTGTGGACCCCCCAAAGTGGGCAAGTCGCTGCTCATTCACT
GTCTCATCAAGTCATTCACCAAACAACCCCTAACCACCCTCAAGGGACCTGTTACTGTTGTC
ACAGGTAAGAAAAGGCGAATCACGTTCATGGAATGCAACAACGATATCAACAGCATGATT
GACTTGGCCAAGATAGCTGATTTAGTTCTGTTACTAATAGATGCGTCGTTTGGCTTCGAGAT
GGAAATATTCGAATTTCTCAACATTTGTCAAGTACATGGTATGCCGAAAATAATGGGCGTC
CTGACGCATCTTGACATGCTGAAGAATACGAAAGCGTTGAAGAAGACGAAAAAAACGCTG
AAACATCGCTTCTGGACGGAGGTGTACGCAGGCGCTAAACTGTTCTACTTGTCTGGCATTCT
GCACAATGAGTATTTGCGGAACGAGGTGAAGAACTTGGGAAGATTCATATCTGTCATGAAG
TTCAGGCCGCTCATTTGGCAGATGACGCATCCGTATCTTTTGGCCGACCGTATGGAAGATCT
GACATCACCAGAAGTGCTGAGACAGAATGACAAGGCGGACCGCACTGTGAGTGTTTATGG
ATATATGCGAGGAATACCTCTCATCAAAAACTCCTCTGTACATATTCCAGGTTGTGGGGATT
TCTTGCTGAAAGATGTGTCGTTTTTGCCCGACCCCTGCCCGCTGCCGGATAAGCTGAAAAAG
CGCGCCCTAGGCGAGAAAGAAAGACTGATTTATGCGCCGTTCTCGGGCGTCGGTGGCATTG
TCTACGATAAGGACGCCGTCTACATTGAACTGGCCGGTAGTCACTCGCATACTAAAAGCAG
TAGTGGGGAGCAACCAGTACGAGATATGGTATCAAACATTCTGGAATCGAAAAATACCCT
CGACTCCAAGATTGCTCAAAGTGAAGTTCAGTTGTTCTCTAATGCTGCACCTATACTGGCTT
CCGAATTCCAGAGTGATACAACTGCCTCGAATCTGCCAGAAGGTATCAGTTTGAAACATGT
GGATGATAACGGGAGACAACGGCGGAAAGTCATCTTCAACGACGAAGAAGACAATATGG
AAGTAGATGGGGATGAGGAGGATGGAGAAGAAGAAGATGATGATGATGAGGGCATAGAA
GACGATAACAGTGACTCGGAATTGAGTAACCAAGAAGATGATGAAGAGGAAGAAGAAGA
AGGTGATGAAAGTGAAGAAGAGGAGGAAGAAGAGGACGGAGAGGAAGAGAAAGGGAAG
ACAAGTAAAACGAGTAAAGTGAAGAAAGCTGATAGCGAGGATGATGATGATGACGATTCC
GAGGAAGAAGATGATGATGATGATGATGATGATTTGATGGAAGACAAGACTGGCTTGAAT

TGGAAATCAAATCTGGCTCAGAAAGCGGCCAACGCTTTCCTAGAGAGACAGGCTGATTCA
GCCAGTTTATGGAAGTTAGTCTATGGAGAGACGAACTACCAAGGGGTGAGAGGAGTGAGG
GGGAAGGAAGAAGAAGACAAGGAAGATGAAGATTTGGAAGAGGGATTATTTAAGATGGT
GAAGCAGAACGAGGATAAGAAGATATTGGAGAAGGATTTGATGGATTTGGAAGATGTCAC
GAAGAAAATTAATAAGGAGCACAGTAGGGACTGGAGTCAAGAGGAGGTGCGTAATACAA
TCAAAGACTGTTTCGTCACTGGGAAATGGAAAAGCTCAGAGGATGCCAGGAAACTACTCA
ATGGGGTTAAAATTGATGATGATGACGATGATGATATCTGTGATGGAGATTTCGAAGATCT
GGAAACCGGAGTAAAACACGAAGGCAAAACGGATGAGCCCACTGATGAAGCTGCCGTAG
ACGATGATAAGTCGAAAGAGAAACTGGCGGAAAAGAAGAAGAAACTGAAAGAGAAATTT
GACGTGGAGTATGACGATGGAAAAAACGGAGGAGATAAGACCTGGTATGACGAATTGAG
GAGGGAAGCCACTGAGCAGGCTGAGTTGAACAGGAAACAGTTCGAGGACATGGATGATTG
GCTGCGAGTTCAGTTGGAGGGGTTCCGCGCCGGCCTCTATGTGCGGATCGAGCTGGATGGA
GTGCCCTGTGAGCTGGTCACCAACTTTGACGCCACCTATCCGCTCATAGTCGGCAGTCTGCA
GCCGGGCGAGGAGAATGTCGGCTTTGTCAAGGCCAGAGTGAAAAAACACCGTTGGTTCGA
GAGAATTCTGAAAACCAATGACCCACTTATTCTATCATTGGGTTGGAGAAGGTTCCAAACA
ATTGCCGTCTATGCAAAACTGGAGGATAATATGAGGCATCGAATGCTGAAGTATACACCCC
AACACATAGCTTGTATGGCGCATTTCTGGGGACCAATCACAAGGTCAGGAACTGGTCTACT
GGCGGTTCAGGATGTAGCTGCTAGACAGTCTGGCTTCCGGATAGCAGCAACGGGAATCGTG
CTGGATACAAACCAGACGACAGAGATAACGAAAAAGCTGAAACTGACCGGAGTGCCTTAC
AAAATCTACAAGAAAACGGCGTTCATCAAGGACATGTTCAACAGTAACCTGGAGGTGGCG
CGCTTCGAGGGCGCCCGCATCAAAACTGTGTCCAACATTCGCGGTCAGATCAAGAAGGCC
GAGAGCAAACCTGAGGGCGCGTTCAGGGCCACTTTCGAGGACAAGATCCAGCTGAGTGAC
ATAGTATTCTGTCGTACCTGGTACAAAGTGGATGTGCCTCGTATGTACAACCCTGTGACGTC
ACTGCTGTTGCCTCCTGAAAGCAAGAACTCTTGGCGAGGCATGAAGACTGTAGGCCAACTC
AAGCGAGAGAAGGGTCTGCACAGTCAGCCTCAGACCGACAGTCTCTATACGGAAATAAAA
CGAGAGCCTGTCGTGTTTAAACCTCTGATTATTCCAAGGCGTCTTCAAAAGGAACTTCCCTA
CAGAATCAAGCCGAAACATCGCCACAATATGAGGAAGAACGCTCCTAAGATAGACAGAGT
GGCTGTTGTCAGGGATGTCGAAGAATCTAAAGTGGCAAGCTTAATGGCCAAACTGAAGTCC
AACTACAAGGCGAAGGTGAGGCAGCAGAAACGCGACACGGCTGCTAGACTGAAAGAGCA
TCGACAGACTCACCACTTGGAGGAAATGCAGCGACAGAGAAAGCGAGCCGATAAAATCA
AAGCTCAACACAGGAAACAGGCCAAAAAACAGGCTCAATCAAGCACCTAA

Primers for *chr11.0845*.

Primer usage	Direction	Primer sequence (5′–3′)	RNAi efficiency (%)
qPCR	F	GACCGGAGTGCCTTACAAAATC	
	R	TCCTCGAAAGTGGCCCTGAAC	
	F	T7-TGATGATGATGACGATGATG	91.40 ± 2.53
	R	T7-CAGACTGCCGACTATGAG	

>chr13.0081

ATGTCTACAACCTTATCTTTGATCAACACAGTATTTCGGTTTGTTTCTGCCCAAAAAACATTA
AAAATTGGGCTTAAAACTTTAGTAAATCCCAATTATTTTGCTGTTGTACGTCATGCTTCATGC
TATAGAGCAGCAGTTCTAAAAGCCGTCAATGAGCCGCTAGTAATCGAAGATGTAAAACAA
GTCAAACTAAGAAAAGGCCAAGTTCGAATAAAAGTCGAGGCATGTGCTGTAAACGTCTCA
GATCTACTGATGTATCAAGGAGTGAGTCCAGTACAACCCAGTCTACCTTTTGTACCAGTTGT
GTTCGTTATGTTCAGGACCAGGTTTATGGCCTATCAAATTTATAATCCGACTCCAGGACTCT
TTCCTACGGTCCTTCAAAGTTTACATGATGTGTGGCGTATCCCCTCCAGCCTGAGCTTCAGT
ACGGCAGCCGGATTGGCTGATGCATATGCTTTGGCACTGCTGGGATTGGCTAGACGAGCCT
GCATTAAGGCCAATAACCTGGTGCTTATCACAACCGGGGTTGGAGGTCTCGGATTGGCTGC
TATTGATGTCGCTTCCAATGTTTATAAAGCTAAGGTAATCGGCATGTGTAAGACAGAGAAA
ACAGGTGAATTACTGCGTCAGAAGGGGGCTTGGAGTGCCATCACTTTCGGAGAGAAAGGA
CTGAAGAAAAAAGTGGCCGAATTAAGTGGAAAACTGGGGGTGGACACTGTTTTTGAGACC

GTGGGTGATGAGGTGTTTAACACTGCTATTCATAGCGTCGCTCATGAAGGCAAAGTGATAG
TGGCTGGATTTGCATCGCAGAAAATTCCTGACATTCCAATGTCGGAATTGTTGACTCTGCCT
TCGTTCAGCCTGATCGGGGTCTCTCTCAGGGACTATAGAGAAACAAATTACAACGTTTACA
GGCAACTAGTCTACGATGTTCTAACACTACACGAACAGGACATCATTAAACCAGTTATTTC
AAAAGAATTCAAGCTAGAGCAAGTGAATGAGGCCCTGAAACATCTGGCCGAAACTGAGAC
TATTGGCAAAATTATACTTACAACTTCTAGTAAAAAGTAG

Primers for *chr13.0081*.

Primer usage	Direction	Primer sequence (5′–3′)	RNAi efficiency (%)
qPCR	F	GAATTGTTGACTCTGCCTTC	
	R	TCCTGTTCGTGTAGTGTTAG	
	F	T7-AATGAGCCGCTAGTAATCG	96.40 ± 1.94
	R	T7-GACATCAATAGCAGCCAATC	

>chrX.0458

ATGGAAACTTACGTTAACCAGCTTCAGAAGTTTCTCAGAGAATCTTTAAATGTAGATGATG
ATGATGATGATATTATTTTTGAATTCAAAAAACCCAAATATCTTCAGTGTTTTGATACTTTTG
GTGTGGATGATCATTCTGAAATTATCTTTATTCGAAAAGAAATAACATATGATGGCTCTGAT
GATCTTAATGGGTCATTATCTACAAACATTTGTCCACCTAAGGACACCTATCGAGAGAGTGT
GTCTGAGTTTGATCTGAGTGGTCAGCCCTTGAACACTGATATGAATGAGTCACTTCTCAGAT
GCGGCACCTCAATCATCCAAAAAGATAAGATTGATGATCATGTTCCATTGTCTGAAATTGA
AGCCAGAAGTATTCTCTCCTACGCTTGCATGCATTTGAAGCCTGTTAAGCCCTTATGGATTC
TGATGGAGCCATGTGGTCAACAGCAAATCATGATTGGTTGCTCGGTTGACGGTCCATGTATT
ACCAGACATGTGCTAAAACTTACAGATGAAGATACATCAGTCGAGGATATGTTGGGCATTC
ACAGAAGTTATATATCACCAAATAGTAAGATTGATACGGAAGTGATGTGCGACTTCACAGT
AACTCAGTTGGGAGAACGAGAAAGTACTGCAAATGAAAATAACGTGGGAAAAATCACTTT
TCAAGTTTTCTGGAGAGAGCCTTCATTCAAAATTCCCATCACCAATTCCTCAATAAAAATGA
ATTTGCATGGAACTGTCGGCTTGCAAGGGAGCGCTGTCCAGATTTTATGGGAACAATTGGA
GCTCCTTCACGGCTATTTCGAGCTGTTGTCCAAATGGAATGACAGTGAATCTTATCCACACG
CTGAGCAGCAGAAATCGGTATTCCTTCCCCATAGGATCTGCAACTCCGAAACAAATGCCCC
CTTATCAGAGAAAATCACAAGCGCCTTAAACTTTTCATATGACTGGTATTCCGGGAAAAAT
AACGAACAAACTCTTACCAGTGTTCTGACGGAACTGAAATCTGGAGAACGTCAGTATTCAG
ATGTTACCGATAGGCTGTGGCTTATCCTAACAGAATGCACCACTCACTGGGAATTGACTAA
CAATATGGCATTCCTATTGCAAGGTGTGAAGAAAGTATCAACAAATACTTATATGAGTGCC
GATAATCATACAAGGCTGGGCAAAGCTATAACGACTGTTAAAAATGACCGGAATGCTGCA
AACCCTCTGTTTCTCTTGAAACCGCTCCAGCTTCTTGTTGAGATTGGCTTGCTGAAACTAAG
GAGAGACTACATCACCATATTCACGGATACTTTACTAGCGAAAACCGAGGCATTAGCAGTA
CCCGAACCTCCAAGCTTCATTTCAATGGATGGCTCATCTTGGTCTGAAACGGTTTCTTCATTG
CTGTGTTGGATGGCAAAAGTTCACGCGGCTTTGGAACTGCTGTGTGTAGCGCACTCTAATCT
GTTGGTGAAGAAAGCAACTATTGTTTCACTGACACGACAAATTTTGGAGAAGTATGTCGGA
TCAAAATCACCCATCCAATCGTTTACTTGGCTCAAAAATAACCCAAATCTATACTTGAGTGC
CAAAATGGACCGACAATCATCAAATATACCGCAAGATCCAAAATATGATAAATGGTTGAT
GAAATTGTCGACCACAGAAAATGATAATCAATATGTGGAAACGAAATTCCTGCGAACCAG
TGAGCCAATGTTTCCAGCTTCAGTCCTTCATATGAGAACAAAGGATGATTGCACCGAAAAT
ACACTGTTATACCTCGAAAATGCAAGCTATACTTTTTCACAGATGACTACCATCTGCAGTGG
ATTAGGAAGTAAGAAAAAATAG

Primers for *chrX.0458*.

Primer usage	Direction	Primer sequence (5′–3′)	RNAi efficiency (%)
	F	GGCACCTCAATCATCCAA	
qPCR	R	GGCTCCATCAGAATCCATAA	
	F	T7-ATCTTATCCACACGCTGAG	90.22 ± 3.35

	R	T7-GTGAATATGGTGATGTAGTCTC	

>chrX.1560

ATGACGGAGATCCGGCAGCGATTCGAAAATCACGAGGAAGAAATCGCCGATCTCGGAGAA
CAGGAGAAACCTGTCAACAAGGAGGAACCGGCACCAGTTGGTGATGAGTTGATGGTTGCC
TCTGGTGAAGGGGACATTGTTTGCAACGATGACGTGCAGCCTGACTTGCAACCCGAGAACG
AGCACATCATGACCTGCGGCGAGAACATGGGAAGTTCCATTTTCGGAAAAGGAAATGATG
AGAAAAAGCCTGAATGTTCTTTGAAGAAAAGAAGATTGGCAGAAGAGGCCGATGCAGAG
AACGAGAAGAGGTCGAGAATTGAGGAGCCAATGGCTACTATGAACCACCATGTTACTGAG
GCACCAGTTGGTGATGAGTTGATGGTTGCCTCTGGTGAAGGGGACATTGTTCACAACGATG
ACGCGCAACCCGAGAACGAGCACATCATGACCCGCGGCGAGAACGAAGAAGAACAAAGA
GATCATGCAGAGATCACATTGCAGGTATCTATAGCACTCCAAAGACTCGGAGATTCAGGAA
GACTTGAGGAGGCTCAACAAGAAGATACAGATGAGGAACATTATGAAGAGGAAGACATT
AATGTCAGCGAAAGTGAAGATGAGGACGACCTTGAGGTTGATGATGATGATGATGATGAT
GATGATGATGATGATGATGAAGACGACGACGAAGAAGACGATGAAGAAGATGAAGATGA
TGATGATGATGATTACAATGTTAAAGATGATGTCAAAAACATAATCCATCGATTTACGTATT
TGCTGGGAAATGATATTAGAAGCTTAATGCTATACCTTTCTATATTTAACGGTTTTGAGAAG
AATACTACTACAGCACTGCACATCAGCGCTTGGCTGGGACATGAGACATTGGCACTTAGAT
TGTTGAAGAAAGGAAGAAATGTTGATGTCAATGCTCGAGATATTGGAGGGCGAACTGCTCT
CCACATGGCTGCCTGGAAAGGCCACAGTGAAATCATTCAAATGCTCCTGGAACACGGCGCT
CACCCGTTGGTCATCAGTCACCAAGCAGCTACTCCTTTGGCGATTGCTATTCAGGAGGGAC
ACAATGACTGCATAAAAATACTTGCGACAGCAGGTGGCTTCGGCGAGGTTCAGCAAGAGA
ATCTAAGCAATTCTGGGATATTGTTTGAAAAGCCCCACCTATGGTGA

Primers for *chrX.1560*.

Primer usage	Direction	Primer sequence (5′–3′)	RNAi efficiency (%)
qPCR	F	GCAGAGAACGAGAAGAGG	
	R	CAGAGGCAACCATCAACT	
	F	T7-GAGGAACATTATGAAGAGGAAG	87.73 ± 1.08
	R	T7-TGCTTGGTGACTGATGAC	

>NlKrt1

ATGAAGGTTCTTGTTTGCACTCTGTTCCTGGCAGCCGTTGTGCTGGCCGATGAGAAGAAAGT
CGATGACGTAGTCGCGGCTGAACCAAAGTCAGATTCTGTGGCCGCTGAGCCAGCAAAGGA
CACCAAGACTGAGAAGAGAGGACTCTATGGACTTGGTTACGGATATGGAGGCGGATACAG
CAGCGGTCTCTCTCACGGTCTTGGATACAGCAGCGGTCTCTCTCACGGTATCGGCTACAGTA
GCGGTCTCTCCCATGGTATCGGCTACAGCTCTGGTTACTCCGGTCTGAGCGGTCTTAGCCAC
GGAGGCTACGGAAGCTATGGATCAGGCTATGGAAGCTACGGATCAGGCTACGGATTGGGC
TACGGATCAGGCGGCTACTCGCACGAAGGCTACGTTAAGGCAGTGACCATCCACAAGGAG
GTCCCCGTGCCACATCCTTACCCTGTACCTGTGCCAGTTGAGAAGCATGTGCCATACCCTGT
CAAGGTCCCAGTCGCTGTGCCAGTCGACAGACCTTACCCTGTTCATGTGCCAAAGCCTTACC
CCGTGCCAGTTGAGAAGCCAGTTCCCTACCCAGTTGAGAAGCCTGTACCTTACCCAGTCAA
GGTTCCAGTGAAGGTACCAGTTGCCCAGCCATACCCCGTCGCCGTTCCCAAGCCCTACCCC
GTGCACATTGAGAAGCCAGTAGCCGTTCCAGTTCCTCAGCCAGTCTACGTTCACAAGCCAG
TTCCCGTTGTCGTCAAGAGTCACGGTTATGGAGGTGGTTATGGAAGCTACGGAGGTTATGG
AAGTGGCTATGGATCTTATGGAGGAGGTCACGGTAGCTACTCAAGCTACGACTCTGGTCTC
AGCTACGGAGGTTACTCCGGTGGATACGACAGCGGTTATGGCAGCTACCACCATTAA

Primers for *NlKrt1*.

Primer usage	Direction	Primer sequence (5′–3′)	RNAi efficiency (%)
qPCR	F	GGACACCAAGACTGAGAAG	
	R	TAGCCGATACCGTGAGAG	

	F	T7-TAAGGCAGTGACCATCCA	93.93 ± 0.83
	R	T7-TAACCGCTGTCGTATCCA	

>NlKrt9

ATGATCCGTATAAAGTTACAGATTTTCGCAGTAGCTCTTCTCGCTCTCTCTGTTCTGGCAGAG
GAGAAGAAAGCCGAGAAGAGAGGAGTTCTGAGCGGACTCCATGGAGCTAACAGCATTGG
AGGAGGAAGTTACGGAGGAGGAGGCTATGGCGGAGGTTACTCCGGGGGTCACGGAGGAG
GTTACAGTCTGGGAGGAGGAGTGAGCAGTTTCGGAGGAGGTCATGGATTCTCAGGAGGTTT
GGGAGGAGGATTGGGAGGTATCGCTTATAGCGGACATGGAGGAGGAGGTTCAGGATTTGG
AGGAGGATCAGGATTCGGAGGAGGACTTGGTGGTCTAGGAGGTGGTCTAGGAGGAGGACT
AGGAGGAGGACTAGGAGGAGGCCTAGGAGGAGGTGCCATCACCGTGAACACAGTCTCCC
AACCAGTACCAGTTCCAGTTCCCCAACCAGTCCCCGTCACAGTTACCCGCACAGTTCCCGT
GCCACAACCCTACCCAGTACCTGTATCTGTTCCTCGCCCAGTAGCCGTTCCAGTTCCTCAGC
CATACCCGGTCACTGTTAACCGTCCATACCCAGTTCCCGTTGAACGTCCCTACCCCGTCAGA
GTTCCCCACCCAGTACCCGTTCCAGTTCCTCACCCCGTTCCAGTGTCAGTTCCCCAGCCGTA
CCCAGTAACTGTTAACCGACCAGTTCCCGTATCAGTTCCTCAGCCGATTGTGGTTCCTCAAC
CAGTTCCAGTGGTGGTGTCTTCGGGAGGCGCTGGAGGTGCTGGAGGTTTCTCGAGTGGATTC
GGGGGCGGACTTGGAGGCGGACTAGGAGGTGGACTTGGAGGCGGTCTTGGGGGCGGTCAC
GGACTTGGAAGCGGACTCGGAAGCGGACTTGGAGCTGGCCTGCTCTCTGGCGGTCACGGTG
GAAGCTACAGCAGTGGAGCACTCCTCTCCGGATATGGAGGTGGTTATGGAGGATACTCAGG
TGCTCTATCCGGTGGACACGGAGGATACTCAGGTGGCCTTTCCAGTGGACATGGAGGATAC
TCAGGTGCTCTATCTGGTGGACACGGAGGATACTCAGGTGGTCTATCCAGTGGACATGGAG
GGTACGTAAGCAGCAGTCTAGGATCATATGGATCGGGATCCTATGGATCAGGATCTTACGG
AGGATCCAAGGGCTACTCAAGTGGAGGTCACGGAGGAGCGACTTCCTACTCGTCGACTACC
TTTGGGCATGGAGGTAGCAGCGGAAGTGGATACGCTTCCGGTTTCAAGGGATATCACTAA

Primers for *NlKrt9*.

Primer usage	Direction	Primer sequence (5′–3′)	RNAi efficiency (%)
qPCR	F	AGGACTAGGAGGAGGACTA	
	R	ATGGCTGAGGAACTGGAA	
	F	T7-CAGCATTGGAGGAGGAAG	98.32 ± 2.47
	R	T7-GGCGAGGAACAGATACAG	

>NlKrt10

ATGGTAAGAGGATGGACTGCCGGGGTCCCGCTCCTGGCACTGCTAGCGGCTACAGCATGGG
CCAATCCCACGCCTGCGCACTTCCACATCCAGGGGCCACACTACACGCACTACCAGTCGCA
GGAGGTCGGCACTCAGCACTACCAGGAGCCCCACCAGGCGCACTACCAGGAGTCGCCGCA
CTACCAGGAGCCACAGGAGTCGCAGCAGCACTACCAGCGACAGCAGCCGCCCCAAGCCGC
CACCCAATTCCACCAGTCCCAGTCCATATCGCAGGAGGTTTTGCAGCCGCAGTATACCAGG
GTGGAATCACCTCCCTATCCTGCAAAAGACTCTCCGAAAGGTCTATCTAAAAAGGCCAACC
ATCAACAGCTGCATCGGTTACAAATGCAGTATTTGCAGGAACTCGAAGCAGATGTTGTCAA
CGCCGTACAGACGAGAGGTATAGCCGAGACAATAAAACTGGAGCAGAAATTCCAGGAAG
AAATCCTAGCTGCAGCCGTTGAGACCCTGACGAAAAACAAGGGAAATCCCGAGAAGCAG
GCGCAGGATTTGAAAGAAATAGAAAATCTTGTGAAGAGGGACAAGAGAGCTGTCGAAGTA
CTCACTACTCTACTAGGATCGAGCAGTGGAAACAAGCTGGGAGGAGGCGGTGCTGGAGGC
GATGATGCCGGCAGCTCTGGCTCGGGGACCATCCTCAACCTCTTGGGTCCACTCTTGGGTAG
CAGTAGTGGAGGGGGTACAGGAGGAGATGGCGGGGATGCTGGATCTGGTTCAGGGTCGAT
TTTGAGTATTGCTGGCCCACTTTTGGGCAGTAGCAGTGGGGGTGGAGGAGGTGGAGGAGTT
GAAGGAGAATCAGCTGGTGGCTCAGATTCAGGATCAATTCTCAACCTAGTTGGGCCTCTTC
TTGGATCAAGCAGTGGTACACAATCAGATGCAGATGGTGTTGGGGAGGGAGGAGGTGGTT
CGAATCTGTTGAGTGTGCTGAGCAGTCTGCTAGGCAGTAGCAGTGGAGGCGGAGGCGGCG
GGGGTGGTGACGCAGAATCAGCTGGAGGTGGTGGCTCAGATTCAGGCTCTATTCTGAATCT

GATTGGACCTCTTTTGGGTAGCAGCAGTGGTGGTAGCTCCAGTGGCGGGGGAGGAGATGTG
GAGGGAGGAGGAGGAGGGGGATCCGATTCAGGCTCCATCCTGAATTTGGTTGGCCCACTTT
TGGGCAGCAGTAGTGGTGGCTCCAGTGGTGGGGGAGGTGATGGGAGTGGCAACGCGGCTG
GTGGATCAGACTCCGGGTCGATTCTGAATCTGGTTGGGCCACTCCTTGGCAGTAGCAGTGG
CGGCAGTTCGTCCGGCGGTGGTGACGCTAGTGGCGGTGGAGGAGGCGGATCAGACTTATTT
GGACTTAGCAACTCAAGCGGAGGAGCAGGTGGATCTGTTAATGGCGCCAAAGTTGGAATT
ATTCAGCAAAAACTACAACTATTTCTAAGGCTAAAGTTCGCAATCTTCACGAAAATCCTCA
ACACTCTGACAGGAGTTTTAGGTTCATCATCAGGTGGAACTCAAGCAGTGCCCATCCATGA
ACTCAAGTATCCATCGTAA

Primers for *NlKrt10*.

Primer usage	Direction	Primer sequence (5′–3′)	RNAi efficiency (%)
qPCR	F	GACCATCCTCAACCTCTTG	
	R	TTCTCCTTCAACTCCTCCA	
	F	T7-ATGGTAAGAGGATGGACTG	92.99 ± 0.89
	R	T7-TTATTGTCTCGGCTATACCT	

© 2020 by the authors. Submitted for possible open access publication under the terms and conditions of the Creative Commons Attribution (CC BY) license (http://creativecommons.org/licenses/by/4.0/).
